# Establishment of new convenient two-line system for hybrid production by targeting mutation of *OPR3* in allopolyploid *Brassica napus*

**DOI:** 10.1093/hr/uhad218

**Published:** 2023-10-27

**Authors:** Hongtao Cheng, Mengyu Hao, Shifei Sang, Yunfei Wen, Yating Cai, Hui Wang, Wenxiang Wang, Desheng Mei, Qiong Hu

**Affiliations:** Department of Rapeseed Genetics and Breeding, Oil Crops Research Institute of Chinese Academy of Agricultural Sciences/Key Laboratory for Biological Sciences and Genetic Improvement of Oil Crops, Ministry of Agriculture and Rural Affairs, Wuhan 430062, China; Department of Rapeseed Genetics and Breeding, Oil Crops Research Institute of Chinese Academy of Agricultural Sciences/Key Laboratory for Biological Sciences and Genetic Improvement of Oil Crops, Ministry of Agriculture and Rural Affairs, Wuhan 430062, China; College of Life Sciences, Henan Normal University, No. 46 Jianshe East Road, Muye District, Xinxiang, Henan, 453007, China; Department of Rapeseed Genetics and Breeding, Oil Crops Research Institute of Chinese Academy of Agricultural Sciences/Key Laboratory for Biological Sciences and Genetic Improvement of Oil Crops, Ministry of Agriculture and Rural Affairs, Wuhan 430062, China; Department of Rapeseed Genetics and Breeding, Oil Crops Research Institute of Chinese Academy of Agricultural Sciences/Key Laboratory for Biological Sciences and Genetic Improvement of Oil Crops, Ministry of Agriculture and Rural Affairs, Wuhan 430062, China; Department of Rapeseed Genetics and Breeding, Oil Crops Research Institute of Chinese Academy of Agricultural Sciences/Key Laboratory for Biological Sciences and Genetic Improvement of Oil Crops, Ministry of Agriculture and Rural Affairs, Wuhan 430062, China; Department of Rapeseed Genetics and Breeding, Oil Crops Research Institute of Chinese Academy of Agricultural Sciences/Key Laboratory for Biological Sciences and Genetic Improvement of Oil Crops, Ministry of Agriculture and Rural Affairs, Wuhan 430062, China; Department of Rapeseed Genetics and Breeding, Oil Crops Research Institute of Chinese Academy of Agricultural Sciences/Key Laboratory for Biological Sciences and Genetic Improvement of Oil Crops, Ministry of Agriculture and Rural Affairs, Wuhan 430062, China; Department of Rapeseed Genetics and Breeding, Oil Crops Research Institute of Chinese Academy of Agricultural Sciences/Key Laboratory for Biological Sciences and Genetic Improvement of Oil Crops, Ministry of Agriculture and Rural Affairs, Wuhan 430062, China

## Abstract

The two-line pollination control system, which usually depends on the utilization of thermosensitive or photoperiod genic male-sterile lines, has been widely used in various crops. However, this system is susceptible to instability issues caused by uncontrollable weather fluctuations. A stable and handy two-line pollination control system is highly desirable in many crop species for heterosis exploitation. *Oxophytodienoic acid reductase 3* (*OPR3*) was proven to be involved in jasmonate biosynthesis. In the present study, CRISPR/Cas9 (Clustered Regularly Interspaced Short Palindromic Repeat) was utilized to mutate two *OPR3* homologs in *Brassica napus*. After two *OPR3* homologs were simultaneously mutated, mutants exhibited complete male sterility, and fertility could be easily restored by exogenous MeJA treatment. Hybrids produced from crosses between the *opr3* sterile lines and normal varieties exhibited heterosis. This new two-line system based on *OPR3* mutation provides higher stability and convenience than traditional systems. By using exogenous MeJA treatment to restore fertility, the system enables more precise control of male fertility transition, which has great potential to significantly contribute to the maneuverable production of hybrid seeds in rapeseed as well as other *Brassica* species crops.

## Introduction

Manipulation of pollination is the principal step in the performance of hybrid breeding. Three-line and two-line systems are the two major systems that have been widely utilized for hybrid production [1, 2]. The three-line pollination control system is mostly based on cytoplasmic male sterility (CMS). Recessive epistatic genic male sterility (GMS) is also a three-line pollination control system, including a male sterile line, a temporary maintainer line, and a restorer in rapeseed. However, three-line systems are usually complicated, labor-consuming, and need sophisticated field management for parental line development and hybrid seed production. The recessive genic two-line system used in many *Brassica* species, including rapeseed, needs to eliminate 50% male fertile plants in hybrid seed production. Development of two-line systems depended on thermosensitive or photoperiod genic male sterility (TGMS and PGMS, respectively) and has widely broadened the utilization of heterosis in rice. The two-line system has wider germplasm resources compared with the three-line CMS system [3]. Several TGMS genes have been isolated in rice and *Arabidopsis*, including genes encoding MYB transcription factors, E3 ubiquitin ligase, a long non-coding RNA, RNase ZS1, leucine-rich repeat receptor-like kinase, and UDP-glucose pyrophosphorylase [4–8]. However, the two-line system of PGMS and TGMS could suffer from the problem of instability due to vulnerability to uncontrollable weather fluctuations [3, 9]. GMS lines with more stable sterility are urgently needed for the application of the two-line system in hybrid seed production [10].

The CRISPR/Cas9 gene editing system has been demonstrated to be a highly efficient and convenient tool to identify multiple homologous GMS genes in polyploid plants [11]. *TMS5* encodes RNase ZS1 and is the most widely used TGMS mutant for the two-line hybrid system in China [7]. Transgene-free *TGMS* lines were developed by knocking down *TMS5* in japonica rice via CRISPR/Cas gene editing within one year [12]. PGMS mutants were generated after targeted mutation of the rice *CARBON-STARVED ANTHERS* (*CSA*) gene in japonica cultivars [13, 14]. A novel male-sterile tomato line with abnormal pollen grains was created by targeted mutation to a particular stamen-related gene known as the *putative strictosidine synthase* gene (*SlSTR1*) [15]. GMS genes have also been explored in bread wheat by targeted mutation of homologous genes of rice and *Arabidopsis*. Triple-homozygous mutants of *TaMs45* and *TaNP1* displayed complete male sterility [16, 17]. Targeted mutation of *OsNP1* homolog in alfalfa also resulted in the generation of a GMS line [18]. Multiple homologous genes required for pollen development and male fertility in maize, including *ZmDFR1*, *ZmDFR2*, *ZmACOS5-1*, and *ZmACOS5-2*, were also functionally studied [11].

Jasmonates (JAs) have been revealed to play a critical role in plant reproduction, including male fertility, seed maturation, and sex determination [19–22]. Loss-of-function of genes that participate in JA biosynthesis caused failure or delay of anther dehiscence and reduced pollen viability, resulting in male-sterile phenotype in *Arabidopsis*[23–27]. These genes include *OPR3*, encoding 12-oxo-phytodienoic acid reductase [23, 24]; *DEFECTIVE IN ANTHER DEHISCENCE1* (*DAD1*), encoding phospholipase A1, a catalyst for the initial step of JA biosynthesis [25]; and *AOS*, encoding allene oxide synthase [28, 29]. COI1 is an F-box protein involved in the ubiquitination pathway in JA signaling [30]. The *coi1-1* mutant is male-sterile and insensitive to JA [31]. OPR3 determines 12-oxophytodienoic acid (OPDA) availability for JA biosynthesis in which OPR3 catalyzes the reductive reaction of jasmonoyl-isoleucine (JAIle) into OPDA [23]. Rice male-sterile lines were generated by knocking out *OsOPR7*, which is the orthologous gene of *Arabidopsis OPR3* [32].

*Brassica napus* is an allopolyploid derived from natural hybridization of *Brassica rapa* and *Brassica oleracea* [33]. To increase the economic value of rapeseed, a multipurpose development and utilization strategy for rapeseed is proposed, with the goal of achieving comprehensive utilization of edible oil, vegetables, ornamental flowers, honey, feed, and fertilizer in the rapeseed industry of China [34]. A recent study explored the nutritional advantages of rapeseed sprouts and provided a reference for the development of rapeseed sprouts into a functional vegetable [35]. The contribution of *B. napus* flowers to the ornamental tourism industry has also been reported and gained much attention for rural revitalization in China [36]. Most of the homologous genes in rapeseed are duplicated and redundant, leading to complexity and difficulty in determining gene function. The CRISPR/Cas9 genome editing system has been successfully utilized to facilitate genetic improvement of important agricultural traits in oilseed rape [37]. In this study, CRISPR/Cas9 was used for the targeted mutation of *OPR3* homologs in *B. napus.* We obtained *opr3* mutants in the spring-type rapeseed variety ‘Westar’ by CRISRP/Cas9. Only the *opr3* mutants with two homologs simultaneously mutated were completely male-sterile and their fertility could be restored by exogenous MeJA treatment. Crosses between *opr3* sterile lines and normal varieties can generate hybrids with heterosis. Our study tried to establish a new two-line pollination control system for hybrid production, which promises to enable the utilization of heterosis in rapeseed production. It will also provide an essential reference for hybrid production in other *Brassica* vegetable crops, as knocking out *OPR3* orthologous genes in these species should also generate a convenient two-line system.

## Results

### *BnOPR3* homolog identification and expression pattern analysis in *B. napus*

**Figure 1:**
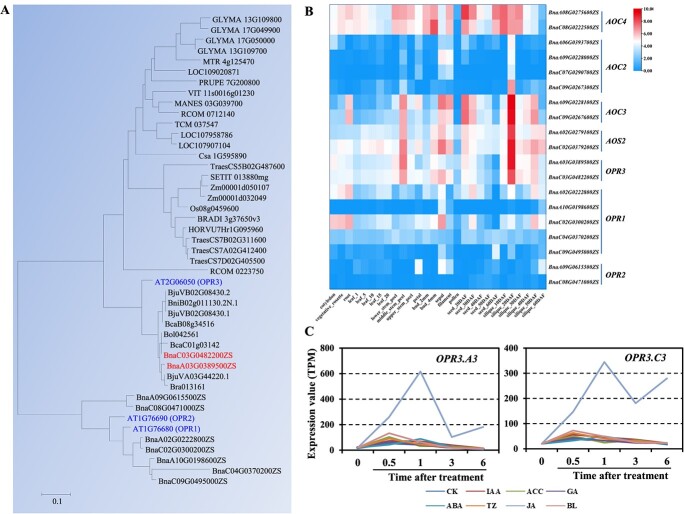
Phylogenetic tree construction and expression pattern analysis of *BnOPR3* in oilseed rape. **A***BnOPR1*, *BnOPR2*, and *BnOPR3* homoeologs were identified in *B. napus* genome. Two homologs of *BnOPR3* are indicated in red color. The tree was constructed by the maximum likelihood method using the ClustalW program with 1000 bootstrap replicates. **B** Tissue expression pattern analysis of *AOC*, *AOS*, and *OPR3* genes involved in JA biosynthesis in oilseed rape. **C** Expression of *BnOPR3* after treatment with MeJA.

Among the three OPRs, only *AtOPR3* has been shown to be involved in JA biosynthesis in *Arabidopsis*. Only one orthologous *OPR3* gene was identified from *B. rapa*, *B. oleracea*, and *B. nigra*, while two or three members were recognized from *B. napus*, *B. juncea*, and *B. carinata* genomes (Fig. 1A). Orthologous genes of *AtOPR1* and *AtOPR2* were also identified from *B. napus* genome and used for sequence alignment. There are quite a few amino acid differences between OPR3 and the other two OPR proteins (Supplementary Data Fig. S1). To gain a better understanding of OPR function, a phylogenetic tree was constructed together with orthologous genes from rice, maize, wheat, tomato, and other species. One *OPR3* clade contained 10 *OPR3* genes and *Arabidopsis OPR3*. Another one contained *Arabidopsis OPR1* and *OPR2* as well as seven *Brassica OPR1* or *OPR2* genes (Fig. 1A). The monocotyledon clade was formed by members from rice, maize, wheat, millet, barley, and *Brachypodium distachyon* (Fig. 1A). The expression level of important genes involved in JA biosynthesis, including *BnAOC*, *BnAOS*, and *BnOPR3*, was checked across different developmental stages using the BnIR database (https://yanglab.hzau.edu.cn/BnIR). The expression level of all these genes was enriched mainly in the developing siliques, stem, and sepal (Fig. 1B). Comparatively low expression was perceived of all these homoeologs in seeds and pollen tissues (Fig. 1B). Lower expression levels of *BnOPR1* and *BnOPR2* were identified in most tissues compared with *BnOPR3* (Fig. 1B). Both *BnOPR3* genes in *B. napus* were highly induced by JA treatment, indicating that *OPR3* genes may participate in JA biosynthesis (Fig. 1C).

### Characterization of target mutation induced by CRISPR/Cas9

**Figure 2:**
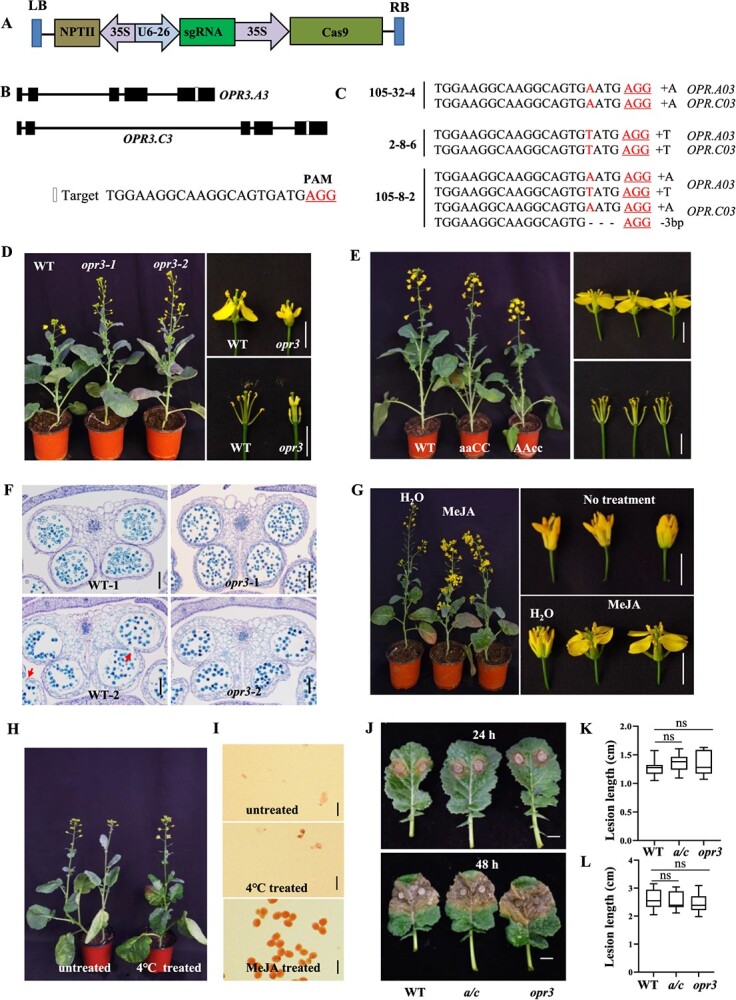
Genotypic and phenotypic analysis of *BnOPR3* mutants and response to MeJA treatment. **A** Schematic diagram of CRISPR/Cas9 construct used for targeted mutation of *BnOPR3*. The construct consisted of a neomycin phosphotransferase II resistance cassette, a Cas9 expression cassette driven by P35S, and one sgRNA driven by the U6-26 promoter from *Arabidopsis*. **B***BnOPR3* gene model with six exons is shown as a box; the white box indicates the sgRNA site. Homozygous mutants 105-32-4 and 2-8-6 were named *opr3-1* and *opr3-2*, respectively. **C** Target sequence analysis of *BnOPR3* homozygous mutant in the *T*_2_ generation. The protospacer-adjacent motif (PAM) is marked in red with underline, and nucleotide indels are marked in red with details at right. **D** Male-sterile phenotype of *Bnopr3* homozygous mutant. *Bnopr3-1* and *Bnopr3-2* represent two independent lines. Scale bar = 1 cm. **E** Phenotype of *Bnopr3* homozygous mutant with only one homolog mutated. aaCC and AAcc represent homozygous mutations of the target gene in the A and C subgenome, respectively. Scale bar = 1 cm. **F** Transverse sections of flower bud from *Bnopr3* homozygous mutant and wild type. 1 and 2 represent buds at the early and late development stages. Scale bar = 100 μm. **G** Phenotype of *opr3* homozygous mutant after spraying with water or MeJA. Scale bar = 1 cm. **H** Male-sterile phenotypes of *Bnopr3* mutant under normal growth condition or treated at 4°C for 1 week before flowering. **I** Pollen grains from *Bnopr3* mutants with or without 4°C treatment were stained with acetocarmine solution. **J** Disease symptoms of *Bnopr3* mutant and wild type after *S. sclerotiorum* infection for 24 h (top) and 48 h (bottom). *a/c*, homozygous mutant with only one homolog mutated; *opr3*, homozygous mutant with both homologs mutated; WT, wild type. **K**, **L** Lesion length analysis after inoculation for 24 h (**K**) and 48 h (**L**).

To further elucidate the role of *BnOPR3*, we designed one single guide RNA (sgRNA) for targeted mutation of both *BnOPR3* homologs in oilseed rape by CRISPR/Cas9 (Fig. 2A and B). The sgRNA located at the fifth exon position can target both *BnOPR3.A03* and *BnOPR3.C03* homologs. However, there are many nucleotide variations of this sgRNA in *BnOPR1* and *BnOPR2* homoeologs. Therefore, this designed sgRNA is specifically for *BnOPR3* homologs. We obtained 34 positive transgenic plants after selection for resistance to neomycin phosphotransferase II. Six plants from 34 positive *T*_0_ transgenic plants were validated to possess nucleotide mutation in both *BnOPR3* homologs (Supplementary Data Fig. S2). Sequencing the PCR products amplified from the target region of the mutants revealed biallelic heterozygous and homozygous mutations at the targeted region. In order to investigate the phenotype of *BnOPR3* mutants, we screened for homozygous mutants in the *T*_1_ generation. Three homozygous *T*_1_ mutants derived from independent *T*_0_ plants with both *BnOPR3.A03* and *BnOPR3.C03* mutated were obtained. Two of these homozygous mutants (105-32-4 and 2-8-6, named *opr3-1* and *opr3-2*) had nucleotide insertion at the target sites resulting in premature termination of BnOPR3 proteins (Fig. 2C). The remaining one (105-8-2) was biallelic with two homozygous allelic variations in each *BnOPR3* homolog (Fig. 2C). In addition, we also screened for homozygous mutants in which only one homolog of *BnOPR3* (aaCC and AAcc) was mutated for further study. Both the *aaCC* and the *AAcc* single mutant of *BnOPR3* came from *T*_0_ heterozygous mutant 105-32.

### Target mutation of two *OPR3* homologs resulting in complete male sterility that could be restored by exogenous MeJA

There was no significant phenotypic difference observed between the homozygous mutants and wild type before flowering. However, an apparent difference in flower buds was identified between mutants and wild type during the flower opening phase, with two independent homozygous mutant lines exhibiting anther indehiscence (Fig. 2D). A large number of pollen grains could be observed on anthers of the wild type, whereas pollen grains were invisible in *BnOPR3* homozygous mutants (Fig. 2D). No pollen grains from *BnOPR3* homozygous mutants were stained by acetocarmine solution, indicating the complete male sterile phenotype (Supplementary Data Fig. S3A–C). Flowers of the mutants were found to be somewhat wrinkled compared with the wild type (Fig. 2D). We then investigated the phenotype of plants with only one homolog mutated. Either *BnOPR3.A03* or *BnOPR3.C03* individual homozygous mutants exhibited normal pollen fertility, as in the wild type (Fig. 2E). Since *BnOPR3* homozygous mutants exhibited anther indehiscence, we then made semithin sections of anthers to determine whether pollen development was normal. The results revealed that pollen developed normally in the *BnOPR3* homozygous mutant, the same as in wild type (Fig. 2F). However, the pollen sac septum had not disappeared in *BnOPR3* homozygous mutants, which resulted in anther indehiscence (Fig. 2F). Only flower buds at stage 12, which corresponds to the final stage before the bud opens, when the petals reach the top of the long stamens in floral development, responded to JA treatment, whereas fertility could not be achieved at earlier and late stages [24]. Therefore, we sprayed *BnOPR3* homozygous mutants with MeJA before the flower buds opened. Pollen grains were visible in the stamens of *BnOPR3* homozygous mutants subjected to MeJA treatment within 24 h (Supplementary Data Fig. S3D and E). Pollen fertility of newly blooming flowers from two *BnOPR3* mutants recovered to ~93.5 and 92.0% after MeJA treatment (Supplementary Data Fig. S3F). By contrast, pollen grains could not be observed in *BnOPR3* homozygous mutants after spraying water as control (Fig. 2G). To detect the stability of anther indehiscence under different conditions, we treated *BnOPR3* homozygous mutants at the bolting stage under 4°C for 1 week. The *BnOPR3* homozygous mutants maintained a stable male sterility phenotype after cold treatment (Fig. 2H and I). After inoculation with *S. sclerotiorum* for 24 h during the flowering period, lesion length on leaves from *BnOPR3* homozygous mutants (1.35 ± 0.2 cm) was similar to that on *BnOPR3* single-mutant (1.37 ± 0.14 cm) and wild-type (1.27 ± 0.15 cm) plants (Fig. 2J to L). There was no significant difference in lesion length between *BnOPR3* homozygous mutants and wild type at 48 h (Fig. 2J–L). We also conducted inoculation with the pathogenic bacterium *Xcc*. Disease symptoms were not different between *BnOPR3* homozygous mutant and wild-type plants (Supplementary Data Fig. S3G).

### Male sterility of *opr3* can be conveniently employed to produce rapeseed hybrids

**Figure 3:**
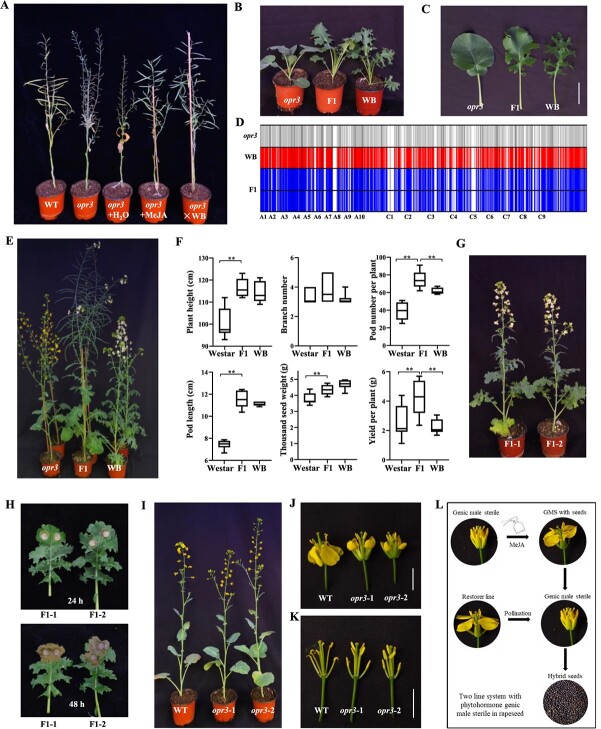
Performance of *Bnopr3* after different treatments and *F*_1_ hybrids. **A** Seed set of *Bnopr3* after spraying with MeJA or water and hybrids after pollination by normal variety. **B**, **C***Bnopr3* mutant and hybrid plants at seedling stage. **D** Genotype analysis of *Bnopr3* mutant, normal variety, and *F*_1_ hybrids by using the *B. napus* 50 K Illumina Infinium SNP array. Gray and red represent the loci in the two parents, respectively. Blue lines represent heterozygous SNP alleles in hybrid plants. **E***Bnopr3* mutant and hybrid plants at flowering stage. **F** Yield traits, including plant height, branch number, pod number per plant, pod length, thousand-seed weight, and yield per plant; comparison between two parents and *F*_1_ hybrid. ^*^<0.05; ^**^, *P* < 0.01; examined by Student’s *t*-test. **G** Flowering phenotype of two hybrids. F1-1, hybrids of ‘Westar’ and WB; F1-2, hybrids of *Bnopr3* and WB. **H** Disease symptoms of two hybrids after *S. sclerotiorum* infection for 24 h (top) and 48 h (bottom). **I***T*_1_ plant from seeds of *Bnopr3* after MeJA treatment. **J**, **K** Flower morphology in *Bnopr3* mutant in *T*_1_ generation and wild type. Scale bar = 1 cm. **L** Schematic diagram of two-line system with phytohormone genic male sterility in rapeseed. Male-sterile plants treated with MeJA can restore fertility and set seeds. Any normal variety can serve as a restorer line to pollinate this genic male-sterile line and generate *F*_1_ hybrids.

To investigate the suitability of the *BnOPR3* mutants for hybrid seed production, we examined plant seed setting after spraying with MeJA. We firstly investigated the pollen viability of *BnOPR3* mutants and wild-type control. The mutants had similar pollen viability after spraying with MeJA compared with wild type, indicating that the mutant was not compromised in this aspect for seed production (Supplementary Data Fig. S3D and E). The *BnOPR3* mutant had a fertilized silique ratio (number of pods with seeds/total number of pods) of ~75% after MeJA treatment, indicating that the mutant was highly responsive to MeJA (Fig. 3A and Supplementary Data Fig.S3H). However, there was no difference in seed setting ratio between the *BnOPR3* mutants with and without water control treatment (Fig. 3A). The exogenous spraying of MeJA was sufficient to restore normal fertility and generate self-pollinated seeds in *BnOPR3* mutants. We then pollinated the *BnOPR3* mutant with a breeding line (WB) with lobed leaves and white flowers. The mutant plants could also maintain a high seed setting rate after cross-pollination (Fig. 3A). As the lobed leaf is an incompletely dominant trait, the hybrid exhibited intermediate leaf morphology (Fig. 3B and C). To further verify the authenticity of the hybrid, a microarray chip was used to examine loci in the parents as well as the hybrid. The results showed that the hybrid exhibited a heterozygous state at all loci tested relative to the two parents (Fig. 3D). Hybrid plants also exhibited white flowers with normal fertility, which is in accordance with white flower color being a dominant trait (Fig. 3E). Meanwhile, the hybrid plants displayed normal seed setting (Fig. 3E). Compared with the two parental lines, the hybrids showed heterosis for various traits, including growth vigor, silique number, and yield per plant (Fig. 3F). Hybrid plants obtained from *Bnopr3* and WB showed similar flowering time, branch number, and plant height to hybrid plants derived from ‘Westar’ and WB (Fig. 3G and Supplementary Data Fig.S4). *Bnopr3* mutants showed traits similar to those of the transformation donor ‘Westar’, including plant height, branch number, and pod number (Supplementary Data Fig. S4). These two *F*_1_ hybrids also exhibited similar disease symptoms after inoculation with *S. sclerotiorum* or *Xcc* (Fig. 3H and Supplementary Data Fig.S5). Therefore, the male-sterile mutant can be well utilized in hybrid production and maintain heterosis in the next generation. The progeny (*opr3-1* and *opr3-2*) from seeds harvested after spraying with MeJA still maintained male sterility (Fig. 3I). The stigma and anther morphology of the mutant progeny plants were identical to those of wild type except for the absence of pollen grains (Fig. 3J and K). Therefore, a two-line pollination control system with phytohormone GMS was established in allotetraploid *B. napus*. The fertility of male-sterile mutant plants can be conveniently restored and seeds can be easily obtained by exogenous MeJA treatment. Any wild-type line can serve as a restorer for generating *F*_1_ hybrids which potentially exhibit heterosis (Fig. 3L).

## Discussion

### New male-sterile line in hybrid production

There are two main types of male sterility—genic male sterility (GMS) and cytoplasmic male sterility (CMS)—which were characterized based on their inheritance mode [39]. CMS and environment-sensitive genic male sterility (EGMS) have been applied in hybrid production for a long time to harness heterosis in rice [40]. Both GMS and CMS systems were used for heterosis utilization in crop species of the *Brassica* genus. Genetic improvement of *B. napus* and other *Brassica* vegetable species is strongly associated with robust hybrid vigor, which is mainly achieved through heterosis breeding. Therefore, an efficient male-sterility system is pivotal for *F*_1_ hybrid seed production. CMS is maternally inherited and can be maintained in a three-line system by crossing with maintainer lines. Fertility of CMS lines can be restored by crossing with restorer lines that carry restorer genes. However, this system is complex and requires sophisticated field experience. The two-line hybrid system offers many advantages in comparison with the three-line system [41]. Three-line hybridization systems including CMS and GMS are usually limited by the availability of restorer or maintainer lines. There are few restorer or maintainer lines that can be applied, making it challenging to obtain sufficient hybrid combinations in three-line system breeding. With a two-line pollination control system based on GMS, nearly all typical fertile varieties can be utilized as restorer lines. However, most naturally occurring GMS genes identified in previous studies are single genes. Many protein-coding genes are classified into gene families and may exhibit partially overlapping functions [42]. As a result, relatively few GMS genes have been identified in polyploid crops since most of the genes are functionally redundant with two or more homologs. It is challenging to simultaneously access multiple gene mutations and generate a GMS phenotype through traditional mutagenesis and map-based cloning [11]. Developing new methods, such as gene editing, to simultaneously mutate multiple genes is important in exploring new GMS genes with redundant function.

### Male sterility regulated by genes involved in jasmonate biosynthesis

JAs regulate plant responses to environmental stresses and also act in many developmental processes, such as plant fertility [43]. Disruption of genes in JA biosynthesis or signaling caused anther indehiscence, defective pollen maturation, and shortened filament elongation [44–46]. Some MYB transcription factors, including MYB21 and MYB24, have been shown to affect JA-mediated male fertility [47, 48]. *Arabidopsis* mutants *dad1* and *opr3* exhibited defective anther dehiscence, filament elongation, and pollen maturation, resulting in male sterility [26]. Disruption of COI1, which is an F-box protein involved in the ubiquitination pathway in JA signaling, also resulted in male sterility [31]. Rice male-sterile lines were created by CRISPR/Cas9 by knocking out *OsOPR7*, which is the orthologous gene of *Arabidopsis OPR3* [32]. In our study, we established a two-line system for hybrid production in oilseed rape based on the *Bnopr3* male-sterile phenotype. *Sclerotinia* stem rot and black rot are two common diseases of *B. napus* caused by *S. sclerotiorum* and *Xcc*. In this study, we found that *BnOPR3* homozygous mutants and the derived *F*_1_ hybrids did not show changed resistance to these two diseases. Meanwhile, the male sterility of *BnOPR3* homozygous mutants was stable after cold treatment. Compared with other types of male sterility, the sterility in the *Bnopr3* mutant is more stable and does not depend on environmental conditions, such as temperature and photoperiod. This male-sterile phenotype can be conveniently restored by MeJA treatment, making reproduction of the male-sterile line feasible. We have also indicated that hybrid seeds can be conveniently produced with any wild-type genotype serving as a restorer line. Therefore, this two-line system with phytohormone GMS has promise for application in hybrid seed production in *B. napus*. Six main cultivated species of the genus *Brassica*, including *B. rapa*, *B. oleracea*, *B. nigra*, *B. napus*, *B. juncea*, and *B. carinata*, are widely utilized for condiments, fodder, and oilseed, or as vegetable or ornamental crops throughout the world [49]. Beside *B. napus*, we identified one to three *OPR3* orthologous genes in the other five *Brassica* species. Knocking out *OPR3*-orthologous genes in these species will also generate a convenient two-line pollination control system, which will greatly facilitate hybrid seed production. Hence the two-line system with phytohormone GMS will have a wide range of applications in *Brassica* species.

### CRISPR genome editing has high potential for generating male-sterile lines

Gene-editing technologies such as the CRISPR/Cas system can introduce precise mutations in specific sites and generate desired phenotypes [50, 51]. Expressing Cas9 with multiple sgRNAs can introduce mutations in multiple sites, which can efficiently facilitate functional dissection of gene family members with redundant roles [42]. *Brassica napus* (AACC) is an allotetraploid derived from hybridization of *B. rapa* (AA) and *B. oleracea* (CC) [33]. Genes in *B. napus* usually contain multiple copies with high sequence similarity, making it unusually difficult to characterize gene function. The CRISPR/Cas9 genome editing system has been applied in oilseed rape to introduce targeted mutations in genes conferring important agronomic traits [22]. Genic male-sterile mutants, including *Tams45* and *Tanp1*, have been created in wheat by using the CRISPR/Cas9 system [16, 17]. The maize *ZmGAMYB* double mutant created by CRISPR/Cas9 technology also displayed male sterility [52]. Multiple homologous genes required for pollen development, including *ZmDFR1*, *ZmDFR2*, *ZmACOS5-1*, and *ZmACOS5-2*, were simultaneously mutated by CRISPR/Cas9 and generated different male-sterile mutants in maize [11]. Consequently, the CRISPR/Cas9 gene editing system has been shown to be a highly efficient and convenient tool for identifying multiple homologous GMS genes. In the present study, we generated novel *BnOPR3* alleles and created a male-sterile mutant line in oilseed rape by targeted mutation of *BnOPR3* involved in the JA biosynthesis pathway. We also ascertained that either the *BnOPR3.A03* or the *BnOPR3.C03* individual homozygous mutant displayed normal pollen fertility, as in the wild type. Thus, the male-sterile phenotype of *Bnopr3* is controlled by two copies with redundant roles. In breeding programs, to obtain male-sterile lines by either targeted mutation or conventional crossing assisted by molecular selection, mutation of both copies should be accomplished simultaneously. Interruption of any functional gene involved in pollen and anther development may result in male sterility. As more plant genes regulating stamen development are identified with the advance of functional genomic studies, genome editing will have high potential to create more genic male-sterility materials.

## Materials and methods

### Plant materials

Transformation donor ‘Westar’ and WB (a breeding line with lobed leaves and white flowers) were propagated at the Oil Crops Research Institute, Chinese Academy of Agricultural Sciences (OCRI-CAAS). Genetic transformation mediated by *Agrobacterium* in rapeseed was mainly carried out according to previously reported methods [37, 38]. Transgenic plants were then transplanted into a growth chamber that provided 20 h of light and 4 h of darkness to enable rapid growth. Flower buds were collected and subjected to paraffin sectioning and staining for analysis.

### Construction of CRISPR/Cas9 vector for *BnOPR3*

Vectors pKSE401 and pCBC-DT1T2 were kindly provided by Professor Qijun Cheng from China Agricultural University. One sgRNA was designed to simultaneously target the coding sequence of two copies of *BnOPR3*. The CRISPR/sgRNA vector was then introduced into GV3101 for genetic transformation.

### Mutation identification of transgenic plants

Genome DNA of transgenic plants was extracted using the CTAB method. T-DNA insertion in the construct was detected by primer U6-26-Pro-F/U6-29-Pro-R (Supplementary Data Table S1). To identify whether *BnaOPR3* was edited, primers specifically targeted to *BnaOPR3* homologs were designed to amplify the fragment-bracketed mutation site (Supplementary Data Table S1). PCR products were used directly to perform Sanger sequencing to identify the target mutation.

### Fertility restoration by exogenous MeJA treatment

*BnOPR3* mutants were treated with exogenous MeJA or H_2_O to study restoration of fertility. Plants were sprayed with 100 μM MeJA solution at the early flowering stage (only about five to eight flower buds opened) one time for each of 3 days, with sterile water used as control to spray the mutants.

### Plant inoculations

Mycelia of *Sclerotinia sclerotiorum* were cultured on potato dextrose agar medium. After culture for ~48 h, agar plugs ~3 mm in diameter were picked out from the edges of colonies and placed onto the surface of plant leaves at the early flowering stage. Pictures were taken and lesion length was determined after inoculation for 24 and 48 h. Lesion length was measured as the diameter of the lesion infected by *S. sclerotiorum*. For *Xanthomonas campestris* pv. *campestris* (*Xcc*) inoculation, *Xcc* strains were cultured in NYGA medium (5 g/L peptone, 3 g/L yeast extract, 20 g/L glycerol, 1.5% agar, pH 7.0). Bacterial cultures were dissolved in water and adjusted to OD600 = 0.4. Leaf tips were clipped at ~1–1.5 cm using scissors dipped in the suspension. Pictures were taken 8 days after *Xcc* inoculation.

### Agronomic trait characterization

*BnOPR3* mutants and wild-type plants were grown in the growth chamber. Plants were photographed at the seedling, bolting, and early flowering stages. More than 10 plants were selected to determine plant height, branch number, silique number, silique length, thousand-seed weight, and yield per plant.

## Supplementary Material

Web_Material_uhad218

## Data Availability

All data supporting the findings of this study are available within the paper and within the supplementary data.
