# Supplementary material for: Establishment of new convenient two-line system for hybrid production by targeting mutation of OPR3 in allopolyploid Brassica napus
Source: Hortic Res. 2023 Oct 27;10(12):uhad218. doi: 10.1093/hr/uhad218 (PMC10699839; doi:10.1093/hr/uhad218)
Supplement: Web_Material_uhad218 [file web_material_uhad218.docx]

**Figure S1.** Sequence alignment of OPR proteins from *Brassica napus* and Arabidopsis. The amino acid sequences: AT2G06050 (OPR3), AT1G76690 (OPR2) and AT1G76690 (OPR1); BnaA09G0615500ZS and BnaC08G0471000ZS were the orthologs of AtOPR2 in the *B.napus* genome; BnaA02G0222800ZS, BnaA10G0198600ZS, BnaC02G0300200ZS, BnaC04G0370200ZS and BnaC09G0495000ZS were the orthologs of AtOPR1 in the *B.napus* genome; Bra013161, Bol042561, BnaC03T0482200ZS, BnaA03T0389500ZS were orthologs of AtOPR3 in the *B. rapa*, *B. oleracea* and *B.napus* genome, respectively.

**Supplemental Table 1.** Primers Used in This Study.

| Primer name | | Forward(5’―3’) | Reverse(5’―3’) |
| --- | --- | --- | --- |
| OPR3t | attgTGGAAGGCAAGGCAGTGATG | | aaacCATCACTGCCTTGCCTTCCA |
| A03OPR3T | GTTCACATTAGCTATGTCATTTT | | CGAACAAGCAGGCCACACAGGAT |
| C03OPR3T | GTTCATATTAGCTATGTCACTGA | | GAAACACGAACAAGCAGGAT |
| U6-26P | TGTCCCAGGATTAGAATGATTAGGC | | AGCCCTCTTCTTTCGATCCATCAAC |


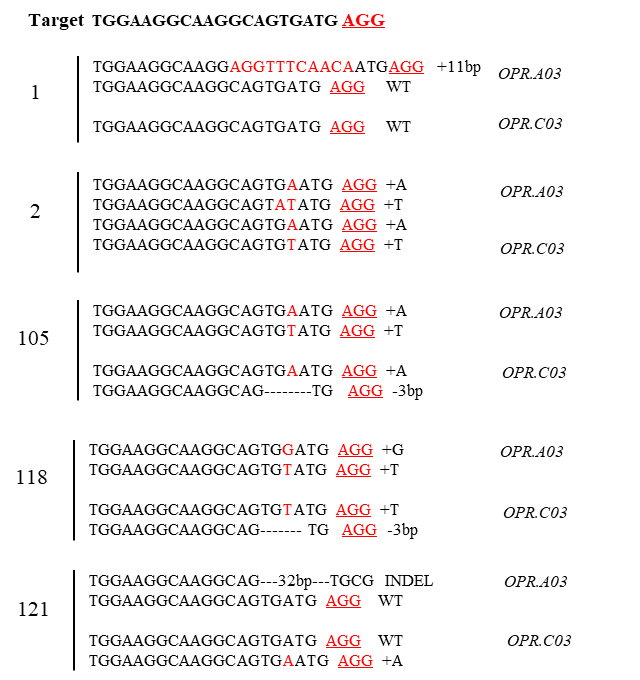


**Figure S2** Target sequence analysis of *BnOPR3* mutant in T_0_ generation. The protospacer adjacent motif (PAM) is marked in red with underline, and nucleotide indels marked in red with details at right.


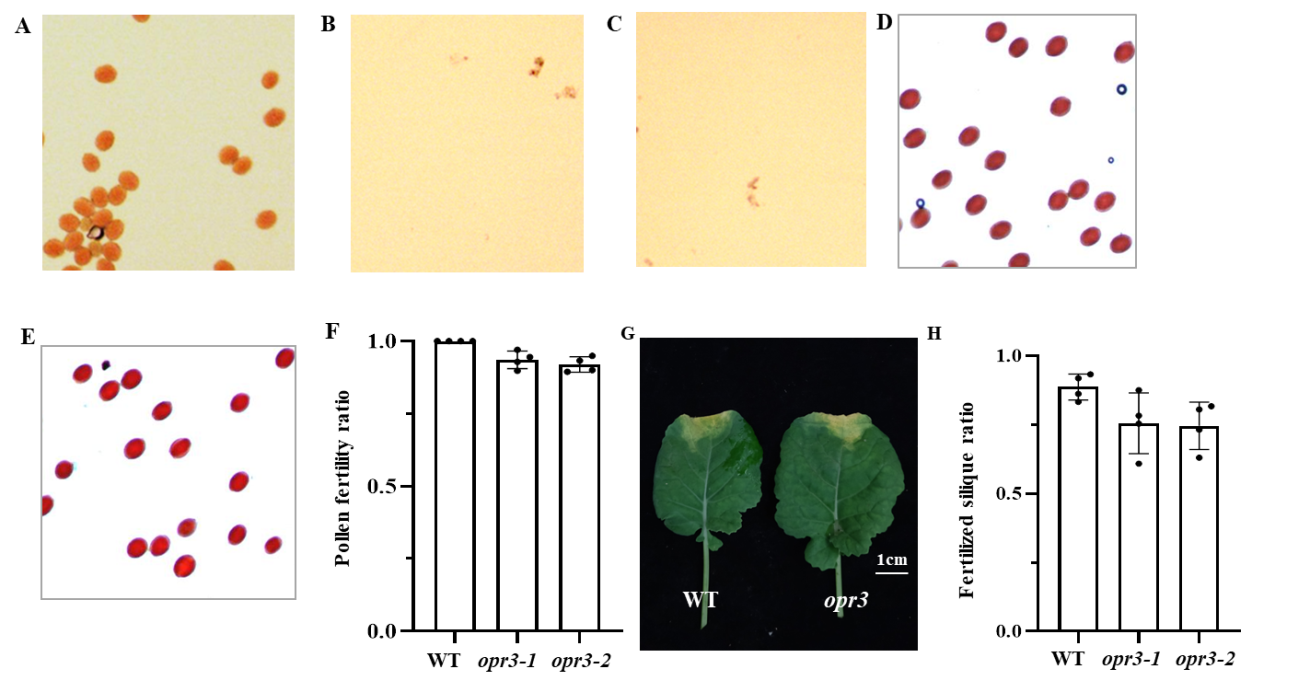


**Supplemental Figure 3.** Pollen stained with Aceto-carmine solution. A and D, pollen from wild type; B, pollen from two *opr3* homozygous mutants. E, pollen from *opr3* homozygous mutant after spray with MeJA. F, Pollen fertility of *opr3* homozygous mutant after spray with MeJA. G, Disease symptom observation after inoculation with *Xcc* of *opr3* mutant and WT. H, fertilized pod ratio of two *opr3* homozygous mutants after treated by MeJA and WT under normal condition.

**
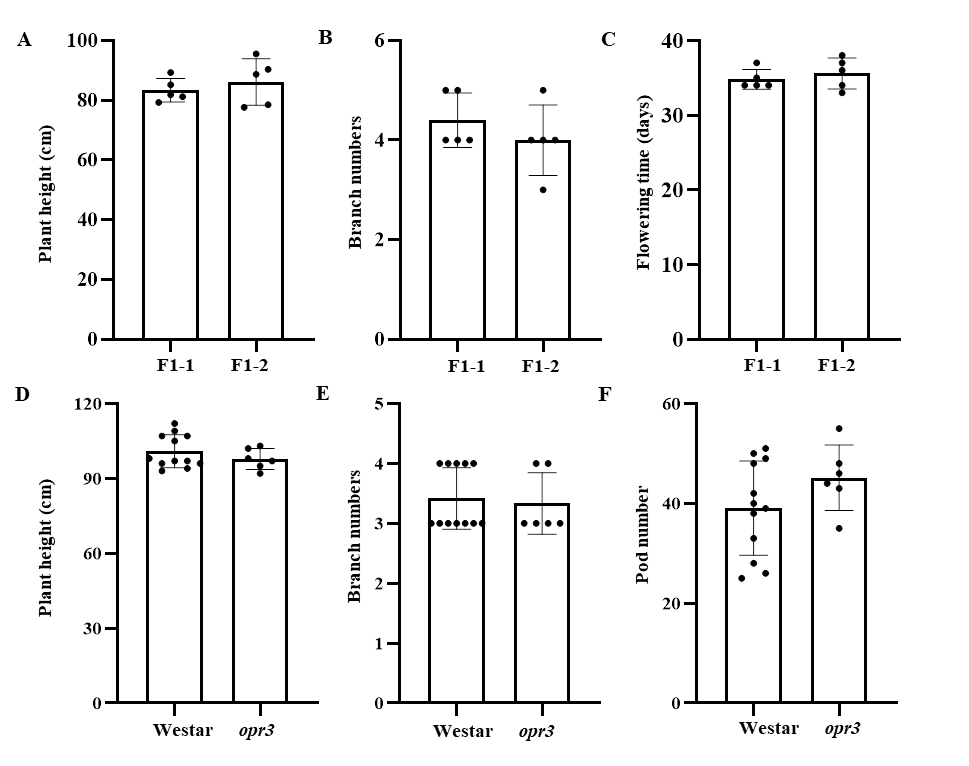
**

**Supplemental Figure 4.** A to C, agronomic traits, including plant height, branch number, flowering time comparison between the two F1 hybrids (F1-1, hybrids between Westar and WB; F1-2, hybrids between *Bnopr3* and WB). D to E, agronomic traits, including plant height, branch number, pod number comparison between the *opr3* and Westar.


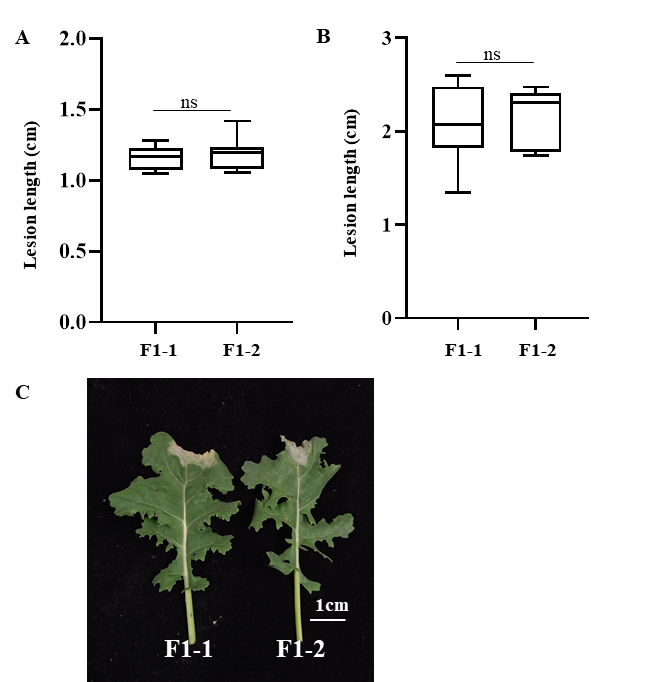


**Supplemental Figure 5.** Disease lesion length measurement in two hybrids after *S. sclerotiorum* or *Xcc* infection. Lesion length was taken at 24h (A) and 48h (B) for *S. sclerotiorum* inoculation. F1-1, hybrids between Westar and WB; F1-2, hybrids between *Bnopr3* and WB. The significant differences are detected by student’s *t*‐test. C, Disease symptom observation after inoculation with *Xcc*. Pictures were taken at 8 days after inoculation.
